# Population size, HIV prevalence, and antiretroviral therapy coverage among key populations in sub-Saharan Africa: collation and synthesis of survey data, 2010–23

**DOI:** 10.1016/S2214-109X(24)00236-5

**Published:** 2024-08-14

**Authors:** Oliver Stevens, Keith Sabin, Rebecca L Anderson, Sonia Arias Garcia, Kalai Willis, Amrita Rao, Anne F McIntyre, Elizabeth Fearon, Emilie Grard, Alice Stuart-Brown, Frances Cowan, Louisa Degenhardt, James Stannah, Jinkou Zhao, Avi J Hakim, Katherine Rucinski, Isabel Sathane, Makini Boothe, Lydia Atuhaire, Peter S Nyasulu, Mathieu Maheu-Giroux, Lucy Platt, Brian Rice, Wolfgang Hladik, Stefan Baral, Mary Mahy, Jeffrey W Imai-Eaton

**Affiliations:** a MRC Centre for Global Infectious Disease Analysis, School of Public Health, Imperial College London, London, UK; b Data for Impact, The Joint United Nations Program on HIV/AIDS (UNAIDS), Geneva, Switzerland; c Johns Hopkins Bloomberg School of Public Health, Baltimore, MD, USA; d US Centers for Disease Control and Prevention, Atlanta, GA, USA; e Institute for Global Health, University College London, London, UK; f Liverpool School of Tropical Medicine, Liverpool, UK; g Centre for Sexual Health and HIV/AIDS Research, Harare, Zimbabwe; h National Drug and Alcohol Research Centre, University New South Wales, Sydney, NSW, Australia; i The Global Fund to Fight AIDS, Tuberculosis and Malaria, Geneva, Switzerland; j Ministry of Health, Maputo, Mozambique; k Data for Impact, The Joint United Nations Program on HIV/AIDS (UNAIDS), Maputo, Mozambique; l Division of Epidemiology and Biostatistics, Department of Global Health, Faculty of Medicine and Health Sciences, Stellenbosch University, Cape Town, South Africa; m Division of Epidemiology and Biostatistics, School of Public Health, Faculty of Health Sciences, University of the Witwatersrand, Johannesburg, South Africa; n Department of Epidemiology and Biostatistics, School of Population and Global Health, McGill University, Montréal, QC, Canada; o Faculty of Public Health and Policy, London School of Hygiene & Tropical Medicine, London, UK; p Sheffield Centre for Health and Related Research (SCHARR), School of Medicine and Population Health, University of Sheffield, Sheffield, UK; q Center for Communicable Disease Dynamics, Department of Epidemiology, Harvard T H Chan School of Public Health, Boston, MA, USA

## Abstract

**Background:**

Key population HIV programmes in sub-Saharan Africa require epidemiological information to ensure equitable and universal access to effective services. We aimed to consolidate and harmonise survey data among female sex workers, men who have sex with men, people who inject drugs, and transgender people to estimate key population size, HIV prevalence, and antiretroviral therapy (ART) coverage for countries in mainland sub-Saharan Africa.

**Methods:**

Key population size estimates, HIV prevalence, and ART coverage data from 39 sub-Saharan Africa countries between 2010 and 2023 were collated from existing databases and verified against source documents. We used Bayesian mixed-effects spatial regression to model urban key population size estimates as a proportion of the gender-matched, year-matched, and area-matched population aged 15–49 years. We modelled subnational key population HIV prevalence and ART coverage with age-matched, gender-matched, year-matched, and province-matched total population estimates as predictors.

**Findings:**

We extracted 2065 key population size data points, 1183 HIV prevalence data points, and 259 ART coverage data points. Across national urban populations, a median of 1·65% (IQR 1·35–1·91) of adult cisgender women were female sex workers, 0·89% (0·77–0·95) were men who have sex with men, 0·32% (0·31–0·34) were men who injected drugs, and 0·10% (0·06–0·12) were women who were transgender. HIV prevalence among key populations was, on average, four to six times higher than matched total population prevalence, and ART coverage was correlated with, but lower than, the total population ART coverage with wide heterogeneity in relative ART coverage across studies. Across sub-Saharan Africa, key populations were estimated as comprising 1·2% (95% credible interval 0·9–1·6) of the total population aged 15–49 years but 6·1% (4·5–8·2) of people living with HIV.

**Interpretation:**

Key populations in sub-Saharan Africa experience higher HIV prevalence and lower ART coverage, underscoring the need for focused prevention and treatment services. In 2024, limited data availability and heterogeneity constrain precise estimates for programming and monitoring trends. Strengthening key population surveys and routine data within national HIV strategic information systems would support more precise estimates.

**Funding:**

UNAIDS, Bill & Melinda Gates Foundation, and US National Institutes of Health.

## Introduction

Key populations, including female sex workers, gay men and other men who have sex with men, people who inject drugs (PWID), and transgender people, experience a higher risk of acquiring and transmitting HIV due to a combination of biological and sociostructural factors, including stigma and criminalisation.^1^, ^2^, ^3^ The Global AIDS Strategy 2021–26 calls for equitable and equal access to HIV prevention and treatment to reduce HIV incidence and end HIV/AIDS as a public health threat by 2030.^4^ Delivering evidence-based HIV services for key populations and monitoring attainment of an equitable HIV response requires robust epidemiological and service engagement indicators, including key population size, HIV prevalence and incidence, and treatment and prevention cascades.

Key population survey data are sparse and less representative of their target population than common general population sampling approaches such as national household surveys or sentinel surveillance. Key population surveys often rely on respondent-driven and venue-based sampling methods,^5^, ^6^ which require strong assumptions to interpret as population-representative. Household survey sampling frames are inappropriate for reaching populations that are mobile and unlikely to disclose risk behaviour due to societal marginalisation and discrimination.^7^, ^8^, ^9^ Key population surveys are often infrequent and commonly restricted to, or disproportionately conducted in, urban areas.

**Research in context**

**Evidence before this study**

Several complementary ongoing initiatives consolidate HIV data on key populations to support programme planning and implementation, global advocacy, and research, including the Key Population Atlas and Global AIDS Monitoring (UNAIDS), databases maintained by the US Centers for Disease Control and Prevention and The Global Fund to Fight AIDS, Tuberculosis and Malaria, and the Global HIV Initiative (Johns Hopkins University). These initiatives include similar data sources, but vary in scope, inclusion criteria, data elements recorded, and linkage to and validation against primary source reports. An incomplete recording of key methodological details limits appraisal and formal evidence synthesis and, therefore, use of data for strategic planning. Many other research studies have systematically reviewed, analysed, and extrapolated key population survey data in sub-Saharan Africa in single countries or across multiple countries. These studies have tended to focus on specific outcomes or population groups of interest, and primarily comprise an appraisal of peer-reviewed literature.

**Added value of this study**

This study was the most comprehensive effort to date to consolidate key population HIV data in sub-Saharan Africa. We analysed more than 3000 observations from 126 key population size estimation studies, 217 HIV prevalence studies, and 62 antiretroviral therapy (ART) coverage studies. We estimated that, across urban populations aged 15–49 years in sub-Saharan Africa countries, a median of 1·65% (IQR 1·35–1·91) of cisgender women are female sex workers, 0·89% (0·77–0·95) are men who have sex with men, 0·32% (0·31–0·34) are men who inject drugs; and 0·10% (0·06–0·12) are transgender women. These population size estimates translated to 3·7 million female sex workers, 1·9 million men who have sex with men, 770 000 people who inject drugs (PWID), and 230 000 transgender women in sub-Saharan Africa who require comprehensive HIV prevention or treatment services. Female sex workers, men who have sex with men, PWID, and transgender women together were estimated as comprising 1·2% of the population aged 15–49 years, but 6·1% of people living with HIV. ART coverage among members of key populations living with HIV increased with total-population ART coverage but was lower for all key populations. We identified large gaps in data availability. Of the four key populations and three indicators analysed in this study (population size, HIV prevalence, and ART coverage) studied, only Mozambique had data for all twelve. Data were particularly sparse for transgender populations and PWID.

**Implications of all the available evidence**

Key populations experience higher HIV prevalence and lower ART coverage across all settings in sub-Saharan Africa than the total population. Extrapolated national estimates provide a foundation for planning appropriate key population-focused services for HIV prevention and treatment in all settings, including those with limited data. However, large data availability gaps driven by discriminatory practices and punitive policies against key populations, inconsistency of existing data, and consequent wide uncertainty ranges around estimates limit the ability of existing data to guide granular programmatic planning and target setting for key population services, and to monitor trends. More consistent surveillance implementation and improved routine surveillance through HIV prevention and treatment programmes for key populations would support monitoring equitable and equal programme access, as outlined in the Global AIDS Strategy 2021–26 developed by UNAIDS, its co-sponsors, and other partners, which aims to end HIV/AIDS as a public health threat by 2030.

Systematic reviews and meta-analyses of population size, HIV prevalence, or antiretroviral therapy (ART) coverage encompassing sub-Saharan Africa have been conducted for female sex workers,^10^, ^11^ men who have sex with men,^12^, ^13^ PWID,^14^, ^15^ and transgender women.^16^, ^17^ Several independent initiatives have been undertaken to consolidate key population surveillance data.^18^, ^19^, ^20^ These efforts aim to monitor the state of the epidemic, evaluate programmes, and make recommendations on key population data reporting and quality thresholds.

We aimed to consolidate and harmonise key population size estimates, HIV prevalence, and ART coverage data from existing databases and describe data availability across countries and over time for each key population. We characterised the relationship between key population and total population HIV indicators and extrapolated key population size estimates, HIV prevalence, and ART coverage data to national-level estimates for mainland countries in sub-Saharan Africa.

## Methods

### Data sources and extraction

For this review**,** we consolidated data from studies conducted between 2010 and 2023 from four existing databases and four systematic reviews among female sex workers, men who have sex with men, PWID, and transgender people.^13^, ^21^ Data among incarcerated people were not included as part of this study. The existing databases were the UNAIDS Global AIDS Monitoring submissions,^18^ UNAIDS Key Population Atlas, the Global Fund to Fight AIDS, Tuberculosis and Malaria surveillance database, and a dataset maintained by the US Centers for Disease Control and Prevention Division of Global HIV & TB Key Population Surveillance Team (appendix 1 p 19). For each observation, where available, we extracted information about the study methodology, study location (country and subnational location), study population gender and age group, central estimate and uncertainty intervals, sample size, and primary source or reference (eg, survey report; appendix 1 pp 20–21).

Source documents were compiled from archives accompanying each database, the Johns Hopkins University Global HIV document repository (not publicly available),^20^ MEDLINE, and Google Scholar, after which we contacted UNAIDS Strategic Information advisors and other HIV programme contacts in each country to seek missing reports. Four authors (OS, RLA, EG, and AS-B) reviewed source documents to identify and remove duplicate observations recorded in multiple databases, validate against sources, and extract missing data elements. During source review, we added observations when additional relevant data were reported, or additional sources were ascertained that were not in the initial databases.

Observations were excluded if key population definition, year, or study area were missing; the study area was non-specific (eg, recorded as “urban areas” or “5 provinces”); estimates were modelled or extrapolated; or data could not be confirmed by primary source review. There were no age group eligibility criteria for inclusion in the analysis. Studies were not excluded or differentiated in our analysis according to study-specific population group definitions based on risk behaviours or time periods (eg, sold sex in the last 6 months *vs* last 12 months; appendix 1 p 4). Transgender men were not analysed in this study due to insufficient data, but observations are detailed elsewhere.^22^, ^23^

This study received ethics approval from the Imperial College Research Integrity and Governance Team (ICREC number 6412027). Regulatory bodies providing ethics approvals or other ethics considerations for included studies are detailed elsewhere.^22^

### Data processing

To compare population sizes across settings, key population size estimates reported as counts were converted to population proportions. Each key population size estimate was matched to a total population denominator^24^, ^25^, ^26^ by age, gender, year, and area, and assigned to one of seven method categories^27^ (appendix 1 p 5). Information on age was missing from nearly all key population reports; missing age ranges were assigned as age 15–49 years, as key population members are predominantly between the ages of 15 years and 49 years.^13^, ^28^, ^29^ Unless gender was specified, PWID were assumed to be men as 90% of people who inject drugs in sub-Saharan Africa are men.^21^ Surveys among transgender people that did not report the gender of the participants, were assumed to be among transgender women because respondents often reported high prevalence of male partners and receptive anal intercourse, and surveys among transgender men in sub-Saharan Africa remain rare.^17^

Key population HIV prevalence was compared with total population HIV prevalence, and ART coverage observations were compared with total population ART coverage, matched by age, gender, year, and first administrative level (henceforth province). Provincial age-specific and gender-specific HIV prevalence and ART coverage were extracted from UNAIDS Naomi subnational estimates for 2022 and projected backwards for 2000–21 parallel to national HIV prevalence and ART coverage trajectories among adults aged 15–49 years.^30^ Only serologically determined HIV prevalence observations were included in the analysis.

Key population ART coverage observations derived from populations with self-reported HIV-positive status or clinic-based recruitment were excluded. ART usage was determined through either self-reporting or laboratory methods via antiretroviral metabolite biomarker or viral load testing. Viral load suppression observations were standardised using a reporting threshold of 1000 copies per mL.^31^ In cases where surveys measured both antiretroviral biomarker and viral load, antiretroviral biomarker was used. Viral load suppression observations were converted to estimates of ART coverage assuming a logit difference of –0·32, which is the average difference from studies that measured both viral load and metabolite-confirmed antiretroviral status (appendix 1 pp 6–7).

### Statistical analysis

We used Bayesian mixed-effects linear regression to model logit-transformed estimates of key population size proportions among the urban total population aged 15–49 years at the provincial level. Separate regressions were estimated for each key population. The model included effects for study method, spatially correlated random effects between neighbouring countries and provinces, and a study-level random effect allowing correlation among key population size estimates proportions from the same study. The study methods were classified as either a non-mapping-based empirical method that estimated total key population size, or Priorities for Local AIDS Control Efforts or mapped estimates, which counted venue-attending or hotspot-attending population size (appendix 1 p 5). Within the empirical method category, method-specific random effects were included to permit deviations from the central estimate. Estimates of national key population size counts were extrapolated using estimates of urban key population size proportions, the proportion of the population living in urban areas,^32^ and the assumed rural-to-urban ratio of key population size. Qualitative data indicate that estimates of key population size proportions are higher in urban areas than rural areas, but no empirical data were available to quantify the rural-to-urban ratio.^33^, ^34^, ^35^ To reflect wide uncertainty in the rural-to-urban ratio, we assumed that estimates of rural key population size proportions were, on average, 40% lower than in urban areas, but with a wide range such that 80% of the time the true value will be between 20% and 60% lower.

For HIV prevalence, we modelled the relationship between logit-transformed key population HIV prevalence and logit total population HIV prevalence (15–49 years). Female sex workers and PWID were modelled separately. Due to limited data, men who have sex with men and transgender women were modelled together but with population type fixed-effects reflecting empirical prevalence difference between transgender women and men who have sex with men.^17^ We modelled survey HIV prevalence observations with a beta-binomial distribution to allow for overdispersion, accounting for additional variation between populations and study designs. The model included fixed-effects for logit population prevalence interacted with region (eastern and southern Africa, or western and central Africa) and province-level, country-level, and study-level random-effects. Country-level and province-level random-effects were spatially correlated.

Primary analysis for key population ART coverage was restricted to diagnostically determined ART usage (either via an antiretroviral biomarker or viral load testing). Logit-transformed key population ART coverage was modelled as a function of logit total population ART coverage in the same year, age group, gender, and province. All key populations were modelled together with random slopes for each key population to enable sharing of information about the overall relationship for groups with very limited data. The observed number on ART was modelled with a beta-binomial distribution with fixed-effects for logit population ART coverage, spatially correlated country-level and province-level random-effects, and study-level random effects.

We conducted sensitivity analyses in which self-reported ART coverage data were also included. For all three models (key population size estimates, prevalence, and ART coverage) in sensitivity analysis, we used ages 15–29 years as the matched total population denominator for men who have sex with men and transgender women regression analyses, and 15–39 years for female sex workers, reflecting the younger median age of survey respondents.^36^, ^37^

We combined national key population size estimates, HIV prevalence, and ART coverage estimates to calculate the number of key populations living with HIV and on ART. 95% credible intervals (CrIs) were generated by combining 1000 posterior samples for each outcome. We calculated 1000 regional median key population size estimate differences from each of the posterior samples of national key population size estimate proportions and calculated 95% CrIs by combining the 1000 regional median differences. The proportion of all people living with HIV associated with each key population was calculated by dividing the number of key populations living with HIV by the total number of people living with HIV aged 15–49 years from 2022 national UNAIDS estimates.^38^

Data were extracted, deduplicated, and validated in Microsoft Excel (version 16.61.1). Statistical analyses were conducted in R (version 4.2.0) using the R-INLA package (version 23.4.24). Further statistical methods have been detailed in appendix 1 (pp 8–12) and the GATHER reporting checklist^29^ can be found in appendix 1 (p 36).

### Role of the funding source

KS and MM are employees of UNAIDS and contributed to the conceptualisation of the study. SAG and MB are employees of UNAIDS and contributed to intepreting the results and editing the manuscript.

## Results

**Figure 1:**
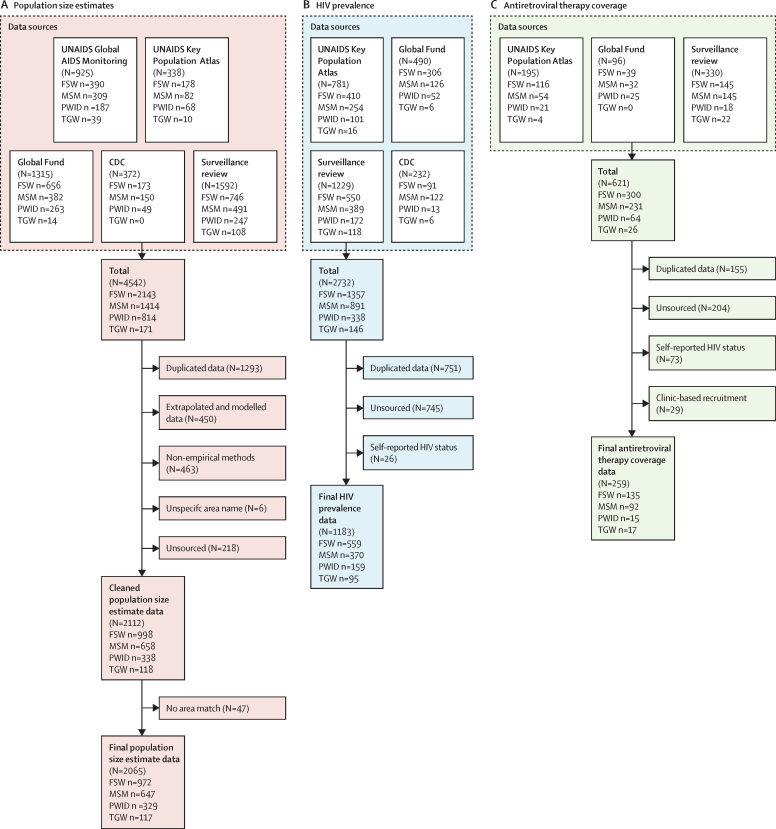
Identified data for key population size estimates (A), HIV prevalence data (B), and key population antiretroviral therapy coverage (C) Each n represents the number of observations in the associated category, with potential observations for multiple subnational locations from the same study in the same country. CDC=US Centers for Disease Control and Prevention. FSW=female sex worker. MSM=men who have sex with men. PWID=people who inject drugs. TGW=transgender women.

4542 key population size estimates conducted between 2010 and 2023 were compiled (figure 1A). Following data cleaning, source document review, and area matching, 2065 observations were extracted from 126 studies (table; appendix 1 p 13). Data were most available for female sex workers (n=972, data from 35 of 39 countries; appendix 1 p 22), followed by men who have sex with men (n=647, 33 countries), PWID (n=329, 23 countries), transgender women (n=117, 13 countries), and transgender men (n=16, two countries). 69% (445 of 647) of urban population size estimate proportions among men who have sex with men were below 1% of the male adult (15–49 years) population (appendix 1 p 14).

**Table:** Availability of population size, HIV prevalence, and antiretroviral therapy coverage data by key population and region during period 2010–23

	**Key population size estimates**		**HIV prevalence**		**Antiretroviral therapy coverage**	
	Data points	Countries with data (n [%])	Data points	Countries with data (n [%])	Data points	Countries with data (n [%])
**Sub-Saharan Africa (39 countries)**						
Female sex workers	972	35 (90%)	559	36 (92%)	135	27 (69%)
Men who have sex with men	647	33 (85%)	370	34 (87%)	92	24 (62%)
People who inject drugs	329	23 (59%)	159	21 (54%)	15	8 (21%)
Transgender women	117	13 (33%)	95	23 (59%)	17	10 (26%)
Transgender men	16	2 (5%)	5	5 (13%)	0	0
**East and southern Africa (18 countries)**						
Female sex workers	450	17 (94%)	236	18 (100%)	85	14 (78%)
Men who have sex with men	291	15 (83%)	127	15 (83%)	45	12 (67%)
People who inject drugs	156	8 (44%)	52	9 (50%)	10	5 (28%)
Transgender women	65	7 (39%)	38	12 (67%)	13	7 (39%)
Transgender men	16	2 (11%)	2	2 (11%)	0	0
**Western and central Africa (21 countries)**						
Female sex workers	522	18 (86%)	323	18 (86%)	50	13 (62%)
Men who have sex with men	356	18 (86%)	243	19 (90%)	47	12 (57%)
People who inject drugs	173	15 (71%)	107	12 (57%)	5	3 (14%)
Transgender women	52	6 (29%)	57	11 (52%)	4	3 (14%)
Transgender men	0	0	3	3 (14%)	0	0

We identified 2732 key population HIV prevalence estimates, from which 1183 data points were extracted from 217 studies after processing (figure 1B). Denominators were reported for 98% (1154 of 1183) of observations. Most data were available for female sex workers (n=559, 36 of 39 countries), followed by men who have sex with men (n=370, 34 countries), PWID (n=159, 21 countries), transgender women (n=95, 23 countries), and transgender men (n=5, five countries).

For ART coverage, 621 observations were identified (figure 1C). After processing, 259 observations were extracted from 62 studies. Denominators were available for 98% (254 of 259) of observations. Data were most available for female sex workers (n=135, 27 of 39 countries), followed by men who have sex with men (n=92, 24 countries), transgender women (n=17, ten countries), and PWID (n=15, eight countries). No data were available for transgender men. 59% (152 of 259) of observations were viral load suppression, 9% (24 observations) were ART metabolite testing, and 32% (83 observations) were self-reported ART usage.

**Figure 2:**
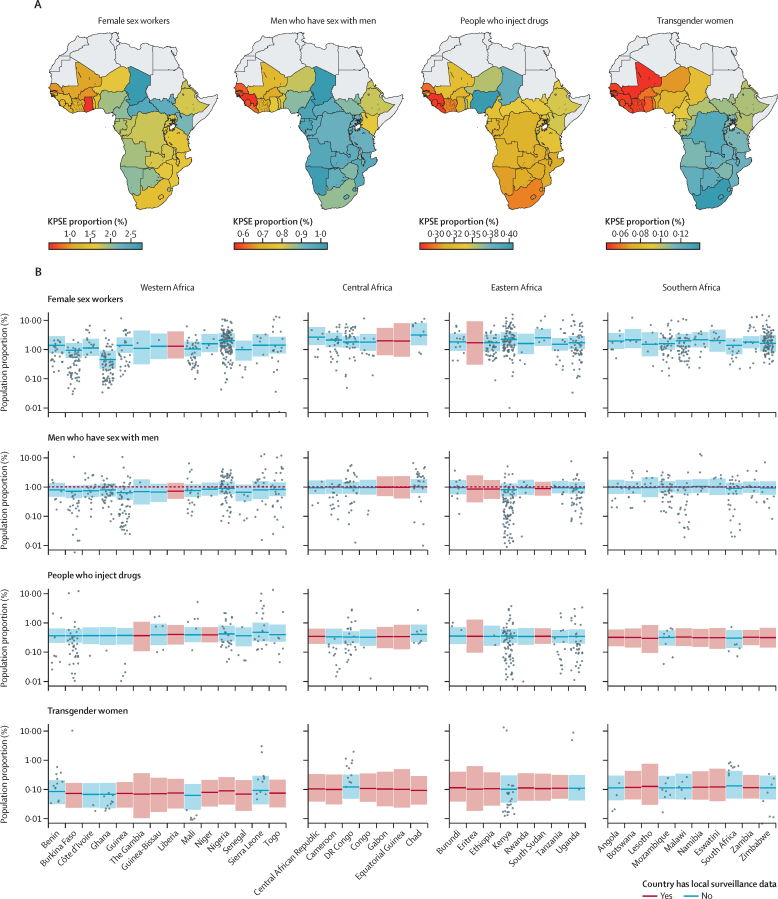
Model estimated urban KPSE proportions for female sex workers, men who have sex with men, people who inject drugs, and transgender women as a proportion of gender-matched adult total population aged 15–49 years (A) Posterior median estimate for each country as a proportion (%). The colour range is different for each key population chloropleth. (B) Posterior median estimate and 95% credible intervals for each country. Points represent observations of subnational key population size estimates proportions that used empirical methods. Countries that had local surveillance data have been shown in blue, and countries informed only by spatial smoothing from neighbouring countries in red. The vertical axis has been shown on log-scale. KPSE=key population size estimate

We estimated that, across countries, a median of 1·65% (IQR 1·35–1·91) of urban cisgender women aged 15–49 years were female sex workers, 0·89% (0·77–0·95) were men who had sex with men, 0·32% (0·31–0·34) were men who injected drugs, and 0·10% (0·06–0·12) were women who were transgender (figure 2). Incorporating the rural-to-urban ratio, median proportions of national total populations were 1·19% for female sex workers, 0·66% for men who have sex with men, 0·25% for PWID, and 0·08% for transgender women. In the sensitivity analysis using the age range of 15–29 years as a denominator for population size estimates among men who have sex with men, 15–39 for population size estimates among female sex workers, and 15–39 years for female sex workers population size estimate, median urban proportions were 1·36% (IQR 1·15–1·42) for men who have sex with men and 1·82% (1·60–2·13) for female sex workers (appendix 1 p 15).

Urban key population size estimate proportions were higher in eastern and southern Africa than western and central Africa among female sex workers (median difference 0·3%, 95% CrI +0·31 (–0·28 to 1·02) and men who have sex with men (0·22 (–0·20 to 0·47) but uncertainty ranges contained zero. Proportions were similar between regions among PWID and transgender women, but key population size estimate data were sparse overall. Population size estimate proportions derived from Priorities for Local AIDS Control Efforts and mapping studies were significantly lower than those derived from other methods among female sex workers (odds ratio 0·58; 95% CrI 0·40 to 0·83), men who have sex with men (0·26, 0·17 to 0·41), and PWID (0·31, 0·19 to 0·53), and lower among transgender women, with the 95% CrIs containing 1 (0·27 to 95% CrI 0·07 to 1·11; appendix 1 pp 16, 23, 28–31).

**Figure 3:**
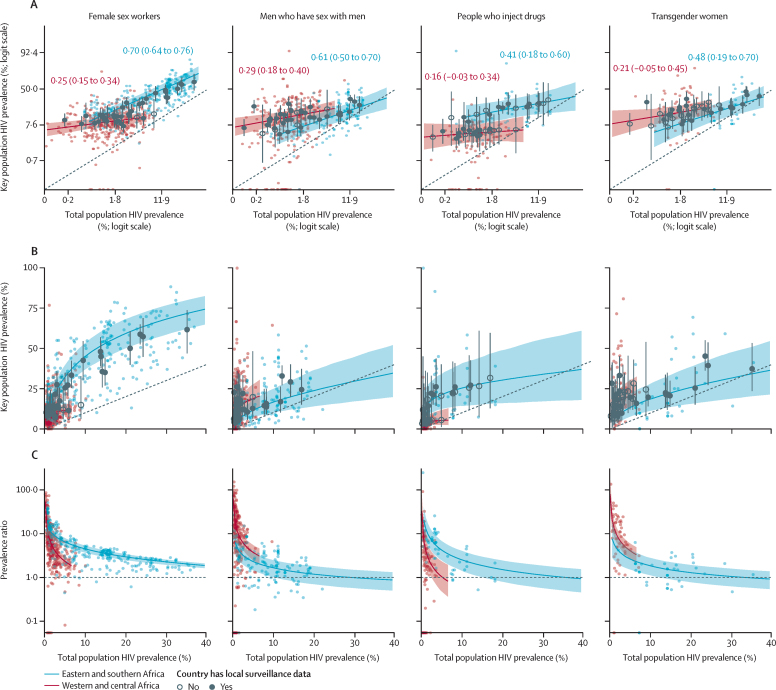
Key population and total population HIV prevalence on the logit scale (A), natural scale (B), and expressed as a ratio of key population and total population prevalence for female sex workers, men who have sex with men, people who inject drugs, and transgender women (C) Coloured points indicate observed key population prevalence plotted against gender-matched, year-matched, and province-matched total population prevalence. The dotted line represents line of equality. Logit-scale regional correlation coefficients and 95% uncertainty intervals shown in coloured text in panel A. Coloured lines and shading represent the regional estimate and 95% uncertainty results. Points represent country estimates (filled for countries with HIV prevalence data and empty for countries without HIV prevalence data) and 95% uncertainty ranges.

Observed HIV prevalence among key populations was consistently higher than matched total population HIV prevalence. Logit key population HIV prevalence in eastern and southern Africa was more strongly correlated with matched logit total population HIV prevalence than in western and central Africa (figure 3). In eastern and southern Africa, total population HIV prevalence was 15%, compared with 53% (95% CrI 45–62) among female sex workers, 21% (12–31) among men who have sex with men, 28% (15–45) among PWID, and 22% (14–34) among transgender women. In western and central Africa, relative patterns were similar, but key population HIV prevalence varied less with total population HIV prevalence than in eastern and southern Africa. HIV prevalence differentials between key populations and total populations were larger when population prevalence was lower (figure 3). Regression results, country-specific estimates, and sensitivity analysis of imputed tested denominators can be found in appendix 1 (pp 17, 24–27, 32).

**Figure 4:**
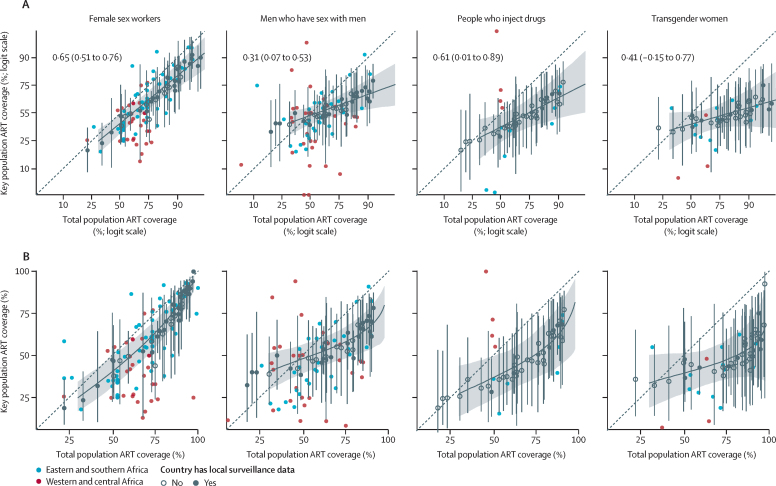
Estimated key population antiretroviral therapy coverage as a function of total population ART coverage on the estimated logit scale (upper) and natural scale (lower) Coloured points indicate observed key population prevalence plotted against gender, year, and province-matched total population ART coverage. Logit-scale correlation coefficients and 95% uncertainty intervals have been shown in part A. The black line and shading represent the estimate for sub-Saharan Africa and 95% uncertainty results. The black points represent country estimates (filled for countries with HIV prevalence data and empty for countries without HIV prevalence data) and 95% uncertainty ranges. The dotted line represents line of equality. ART=antiretroviral therapy.

ART coverage among female sex workers, men who have sex with men, PWID, and transgender women was correlated with gender-matched population ART coverage, but with wide heterogeneity in relative ART coverage across studies (figure 4). At 80% population coverage, predicted ART coverage was 11% lower (95% CrI 19–4) than total population coverage among female sex workers, 19% lower (30–10) among men who have sex with men, 25% lower (41–11) among PWID, and 13% lower (43–18) among transgender women (appendix 1 pp 27, 33).

In the sensitivity analysis including a fixed-effect for ART measurement method, self-reported ART coverage was lower than laboratory-determined ART, but with a wide 95% CrI spanning no difference (OR 0·73, 95% CrI 0·47–1·15; appendix 1 pp 18, 27). Assuming that surveys among men who have sex with men represented those aged 15–29 years or that surveys of female sex workers represented those aged 15–39 years minimally affected HIV prevalence and ART coverage estimates (appendix 1 p 35).

**Figure 5:**
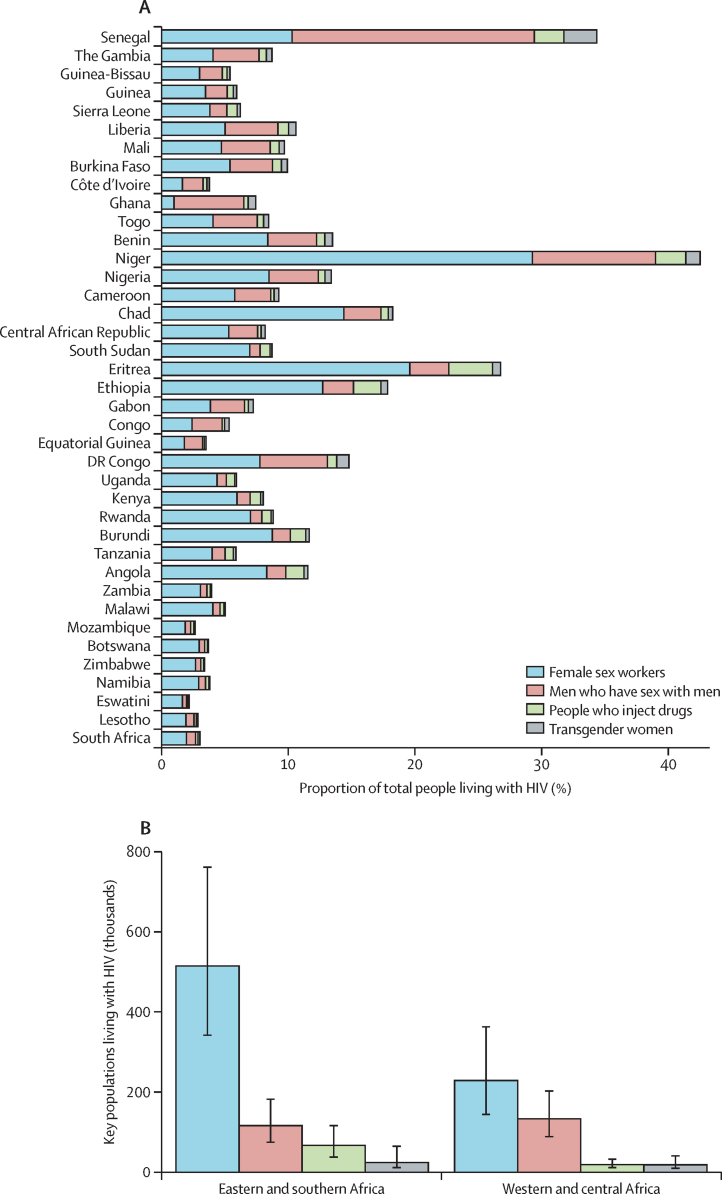
(A) HIV-positive key population members as a proportion of all people with HIV aged 15–49 years. (B) Estimated number of key populations living with HIV in eastern and southern Africa, and western and central Africa Countries are ordered geographically.

Across sub-Saharan Africa, female sex workers, men who have sex with men, PWID, and transgender women combined represented 1·2% (95% CrI 0·9–1·6) of the population aged 15–49 years (3·7 million female sex workers, 1·9 million men who have sex with men, 770 000 PWID, and 230 000 transgender women), but 6·1% (95% CrI 4·5– 8·2) of people living with HIV aged 15–49 years. In eastern and southern Africa, these figures were 1·3% (0·9–1·8) of the population compared with 4·9% (95% CrI 3·4–6·9) of people living with HIV and, in western and central Africa, 1·2% (0·9–1·7) of the population with 11·7% (8·3–16·5) of people living with HIV. Female sex workers were 4·0% (95% CrI 2·7–5·7) of people living with HIV in sub-Saharan Africa (750 000 people living with HIV, 95% CrI 520 000–1 100 000), men who have sex with men were 1·4% (0·9–2·0) or 250 000 people living with HIV (170 000–380 000), PWID were 0·5% (0·3–0·8) or 89 000 people living with HIV (50 000–150 000), and transgender women were 0·2% (0·1–0·5) or 44 000 people with HIV (20 000–100 000; figure 5).

## Discussion

Data from 273 key population surveys provided consistent evidence across sub-Saharan Africa of higher HIV prevalence and lower ART coverage among key populations. We estimated that there are 3·7 million female sex workers, 1·9 million men who have sex with men, 770 000 PWID, and 230 000 transgender women in sub-Saharan Africa who require comprehensive HIV prevention or treatment services. HIV prevalence was estimated to be 21% among female sex workers, 14% among men who have sex with men, 11% among PWID, and 20% among transgender women. These were 4·6, 5·9, 4·6, and 4·4 times higher than adult population prevalence, respectively, with larger relative differences when population prevalence was lower. Together, key populations constituted 1·2% of the adult population in sub-Saharan Africa but 6·1% of people with HIV. ART coverage among key populations increased with total population coverage but lagged behind at high population ART coverage levels. However, there was wide uncertainty about estimates aggregated within countries and across sub-Saharan Africa.

This study has highlighted major data gaps and challenges interpreting existing data. Across the 12 combinations of three indicators and four key populations analysed here, on average, countries had data for seven combinations (appendix 1 p 22). Data gaps are larger when restricting to the highest quality and most recent data. These gaps impede planning for equitable and universal access to HIV services and will persist without firm commitments to collect key population survey data. Implementation of high-quality surveys will also require addressing entrenched discriminatory practices and punitive laws and policies against key populations.^3^, ^39^, ^40^, ^41^ In settings with limited data, our extrapolated estimates can be a foundation with which to guide future surveillance priorities, stimulate in-country data review and use, and estimate HIV epidemic indicators. Future efforts should especially focus on strengthening surveillance for men who have sex with men, PWID, and transgender people, and supporting use of HIV service delivery data for all key populations while ensuring the safety and protection of marginalised individuals and their communities.^3^

Previous key population reviews and meta-analyses^11^, ^15^, ^42^, ^43^ relied on systematic searches of peer-reviewed literature. Our initial sources were existing databases, primarily populated by surveillance teams in each country or implementing organisations, which resulted in inclusion of a larger body of grey literature, such as survey reports, which are infrequently indexed by peer-reviewed databases.^17^ However, many observations from initial databases could not be validated in source documents and were excluded from analysis, which in some countries excluded the only reported data. Demand for total population HIV estimates in sub-Saharan Africa has promoted high-quality comparable surveillance methods, documentation, and dissemination. Review of key population data by national HIV surveillance teams was introduced into the UNAIDS sub-Saharan African estimates process in 2022, providing context for existing surveys, identifying missing sources, and adding new data. Incorporating review into the UNAIDS estimates process, alongside ongoing key population data initiatives and concerted advocacy efforts that ensure public dissemination, will improve the foundation for HIV surveillance among key populations and promote parity for key populations within national HIV data and strategic information.

Systematically collated data on key population size, HIV prevalence, and ART coverage are essential inputs for mathematical modelling analyses that guide optimal HIV resource allocation. Such studies consistently show that fully meeting the HIV prevention and treatment needs of key populations would be impactful on reducing overall population incidence due to network effects of averting further onward transmission over time and to non-key-population partners.^44^, ^45^ Model-based counterfactual indicators that capture these impacts, such as the transmission preventable attributable fraction, are a better basis for intervention prioritisation than simple population proportions reported in survey data.^44^, ^46^, ^47^

Our estimate that 0·29% of men in sub-Saharan Africa injected drugs was lower than estimates from Degenhardt and colleagues^21^ of 0·38%, although uncertainty ranges overlapped, which could reflect incorporating the urban-to-rural ratio in national population size estimate extrapolation in this analysis. We estimated that 1·2% of women aged 15–49 years sold sex, similar to the 1·1% estimated by Laga and colleagues.^48^ Our finding of heterogeneous HIV prevalence ratios between men who have sex with men and the total population was consistent with Hessou and colleagues,^42^ particularly in western and central Africa where HIV burden in neighbouring countries can vary considerably. Our estimate of 57% ART coverage among men who have sex with men in 2022 was lower than 73% from a meta-analysis by Stannah and colleagues^12^ of the studies conducted up to 2022, with overlapping uncertainty. This difference could be due to exclusion of ART coverage using self-reported HIV status in this analysis.

Population size estimates measured in the same population at different times or with multiple methods were highly heterogeneous, limiting data use for establishing meaningful programmatic targets or monitoring temporal trends. Very large uncertainty ranges around population size and modelled estimates of people living with HIV were consistent with findings of other statistically summarised and extrapolated population size estimates from multiple sources.^49^, ^50^, ^51^, ^52^ Non-existence of rural key population size estimates to empirically inform rural–urban population proportions contributed to five-fold or greater relative uncertainty in extrapolated national estimates. Moreover, quality assessments of individual key population size estimates were not conducted as part of this analysis. Both systematically improving and evaluating study-specific implementation quality and methodological assumptions could reduce heterogeneity and increase the use of key population size estimates data.^53^, ^54^, ^55^, ^56^, ^57^

72% of observations and 38 of 39 national modelled key population size estimates among men who have sex with men were below the 1% of the male adult (15–49 years) population that UNAIDS and WHO recommended in 2020 as a minimum population proportion.^19^ The minimum threshold was specified to ensure that men who have sex with men were not overlooked due to lack of data. Variation in observed population size could reflect the ability to engage in sexual behaviours or surveys, affected by legal and social environment and other factors, rather than differences in distribution of sexual preference. It is also uncertain whether men recruited in surveys, who are disproportionately young,^12^, ^13^ are representative of the total men who have sex with men population. Specifying a minimum 1% threshold could result in unrealistic service targets for the population. The threshold should be reconsidered with the wider range of population size and stigma data now available, aligned with risk-based approaches to quantifying sex work and injecting drug use. Study-specific quality assessments will be important in producing accurate key population size estimates given stigma experienced by men who have sex with men in many settings.

Integrating key population programme data within key population estimates should be a priority to improve the use of surveillance data to guide programmatic response and monitor trends and subnational variation.^58^ Triangulating routine HIV testing and treatment data with biobehavioural survey data could help disentangle programmatic double counting, coverage of key population-specific services, and accounting for linkage to care in the estimation of ART coverage and viral load suppression.^8^, ^57^, ^59^ Future surveillance should consider moving beyond strictly defined key population definitions to reflect accurately the heterogeneous risks among individuals in overlapping risk environments, including cisgender men who have sex with men and transgender women who sell sex, and female sex workers and men who have sex with men who inject drugs.^58^, ^60^, ^61^

Several limitations should be considered when interpreting these data and estimates. First, surveys used a range of key population definitions which limited comparability, although there was substantial overlap in risk behaviour definitions and inclusion criteria (appendix 1 p 5). Second, lack of explicit geographical survey sampling frames made it challenging to reflect the catchment population for a given survey in matched denominators, particularly given the mobility of key populations.^62^ Inaccurate denominators likely exacerbate the observed heterogeneity in key population size estimates proportions (figure 2), and are a major limitation for extrapolating key population size estimates for programmatic planning. Going forward, specification of population catchments and careful data quality assessments to determine study inclusion and interpretation will improve future data use. Third, sample size or standard errors and age ranges were omitted from most population size data entries, gender was not specified for most observations among PWID, and a small number of observations were missing denominators for numbers tested. These omissions limit the ability to statistically weigh, age-standardise, or gender-match data in model extrapolations, although sensitivity analyses indicate little effect on conclusions. Fourth, data were insufficient and too heterogeneous to estimate time trends in population proportions, HIV prevalence, or ART coverage. Application of standardised recruitment methods, population definitions, and indicator reporting in consistent locations will improve monitoring of trends. Finally, this study did not include people in the criminal justice system, and other incarcerated people, and future studies should look to expand data synthesis and analysis to this key population.

In conclusion, key populations across sub-Saharan Africa experience disproportionate HIV burden and have lower antiretroviral therapy coverage. Consolidated key population data and synthesised estimates provide a basis for key population programming in all countries, including those with limited locally available data. Despite increasing focus on key populations in HIV/AIDS strategies in sub-Saharan Africa, large data gaps remain and estimates are highly uncertain. New surveillance strategies, improved use of routine data, and more consistent surveillance implementation are required to furnish more precise estimates and trends for programme planning and support the monitoring of equitable and equal access to HIV prevention and treatment programmes outlined in the Global AIDS Strategy 2021–26.

### Contributors

### Equitable partnership declaration

### Data sharing

Data extracted and the code to reproduce the analysis are available at https://zenodo.org/records/10838438 and can be explored at https://shiny.dide.ic.ac.uk/kp-data.

## Declaration of interests

SB has received funding from the US National Institutes of Health (NIH). FC has received funding from the Wellcome Trust, the Medical Research Council, NIH, Unitaid, and the Bill & Melinda Gates Foundation. LD has received untied educational grants for the study of new opioid medications in Australia from Indivior and Sequirus. EF has received funding from the UK Research and Innovation Medical Research Council, the Royal Society, and the Centre for Sexual Health and HIV/AIDS Research Zimbabwe. JWI-E acknowledges funding from UNAIDS, NIH, the Gates Foundation, UK Research and Innovation, and BAO Systems, and has received support to attend meetings from UNAIDS, the South African Centre for Epidemiological Modelling and Analysis, the International AIDS Society, and the Gates Foundation. KR and MM-G have received support to attend meetings from UNAIDS. JS has received funding from UNAIDS. OS has received funding from UNAIDS. All other authors declare no competing interests.

## Supplementary Materials

Supplementary appendix 1

Equitable Partnership Declaration
