# Supplementary material for: Population size, HIV prevalence, and antiretroviral therapy coverage among key populations in sub-Saharan Africa: collation and synthesis of survey data, 2010–23
Source: Lancet Glob Health. 2024 Aug 14;12(9):e1400–12. doi: 10.1016/S2214-109X(24)00236-5 (PMC11345451; doi:10.1016/S2214-109X(24)00236-5)
Supplement: Equitable Partnership Declaration [file mmc2.pdf]

THE LANCET
Global Health

Supplementary appendix 2

This Equitable Partnership Declaration (EPD) was submitted by the authors, and we reproduce it as supplied. It has not been peer reviewed. *The Lancet*'s editorial processes have not been applied to the EPD.

Supplement to: Stevens O, Sabin K, Anderson RL, et al. Population size, HIV prevalence, and antiretroviral therapy coverage among key populations in sub-Saharan Africa: collation and synthesis of survey data, 2010–23. *Lancet Glob Health* 2024; **12:** e1400–12.

Equitable Partnership Declaration questions

This Equitable Partnership Declaration is a statement being published online alongside papers at *The Lancet Global Health*, as a separate appendix, to allow researchers to describe how their work engages with researchers, communities, and environments in the countries of study. This is part of our broader goal to decolonise global health, handing control and leadership of research to academics and clinicians who are based in the regions of study, and to affected communities.

Please answer all questions with as much detail as possible, noting that all included information will be published open-access and it will be freely available online to all who wish to read it. If a question does not apply to your study, please state "Not applicable".

The format of and questions in this statement are currently in a pilot phase. Please email Dr Liam Messin (Liam.Messin@lancet.com; deputy editor) and Dr Kate McIntosh (Kate.McIntosh@lancet.com; senior editor) with any feedback, particularly if you find any questions unclear.

Researcher considerations

1. Please detail the involvement that researchers who are based in the region(s) of study had during a) study design; b) clinical study processes, such as processing blood samples, prescribing medication, or patient recruitment; c) data interpretation; and d) manuscript preparation, commenting on all aspects. If they were not involved in any of these aspects, please explain why.

This question is intended for international partnerships; if all your authors are based in the area of study, this question is not applicable.

This should include a thorough description of their leadership role(s) in the study. Are local researchers named in the author list or the acknowledgements, or are they not mentioned at all (and, if not, why)? Please also describe the involvement of early career researchers based in the location of the study. Some of this information might be repeated from the Contributors section in the manuscript. Note: we adhere to ICMJE authorship criteria when deciding who should be named on a paper.

**a) Study design:**<br>No researchers based in the region of study were involved in study design
**b) Clinical study processes:**<br>Not applicable
**c) Data interpretation:**<br>Researchers based in the region of study, and researchers based outside of the region of study but who have conducted primary data collection studies in the region of study, were involved in interpreting the data, developing regression analyses, and reviewing final estimates.<br><br>All researchers involved in the development of the paper have been included as co-authors
**d) Manuscript preparation:**

The first draft of the manuscript was prepared by researchers outside the region of study. Multiple drafts of the manuscript were reviewed by all co-authors, including those in the region of study.

2. Were the data used in your study collected by authors named on the paper, or have they been extracted from a source such as a national survey? ie, is this a secondary analysis of data that were not collected by the authors of this paper. If the authors of this paper were not involved in data collection, how were data interpreted with sufficient contextual knowledge?

   The Lancet Global Health *believe contextual understanding is crucial for informed data analysis and interpretation.*

   This is a secondary analysis of over 250 key population surveys. Of our 27 authors, eleven have been involved in primary data collection in over 40 studies used within this analysis, encompassing all four key populations analysed in this paper. Their contributions during data analysis and critical review of the manuscript provided the knowledge required for context-appropriate data interpretation and discussion. Other co-authors work closely through their primary employment with national HIV programmes and key population community organizations on priorities, trends, programme strategies and implementation of key population programmes and surveys. This expertise and experience was also critical for guiding study design and interpreting results of this analysis.

3. How was funding used to remunerate and enhance the skills of researchers and institutions based in the area(s) of study? And how was funding used to improve research infrastructure in the area of study?

   Potentially effective investments into long-term skills and opportunities within institutions could include training or mentorship in analytical techniques and manuscript writing, opportunities to lead all or specific aspects of the study, financial remuneration rather than requiring volunteers, and other professional development and educational opportunities.

   Improvements to research infrastructure could be funding of extended trial designs (such as platform trials) and use of master protocols to enable these designs, establishment of long-term contracts for research staff, building research facilities, and local control of funding allocation.

   Skills:

   No funding for this study was directed towards the areas of study

Research infrastructure:

No research infrastructure was funded as part of this study.

4. How did you safeguard the researchers who implemented the study?

   Please describe how you guaranteed safe working conditions for study staff, including provision of appropriate personal protective equipment, protection from violence, and prevention of overworking.

   This study was a secondary data analysis conducted as a desk review. There were no safeguarding concerns for implementing researchers.

Benefits to the communities and regions of study

5. How does the study address the research and policy priorities of its location?

   How were the local priorities determined and then used to inform the research question? Who decided which priorities to take forward? Which elements of the study address those priorities?

   The objectives of this study were derived from a request from UNAIDS to review key population survey data in sub-Saharan Africa such that key populations could be integrated into Spectrum modelling tools used by national HIV programmes to create their annual HIV estimates. The request was received via the UNAIDS Reference Group on Estimates, Modelling, and Projections. The UNAIDS Reference Group is a group of epidemiologists, public health professionals, statisticians, and mathematical modellers that provides technical advice to UNAIDS in support of the HIV estimates process (www.epidem.org).

   A technical expert meeting was convened focusing on key population epidemiology in sub-Saharan Africa, following which the Reference Group recommended (1) key population study data should be collated and validated against primary sources; and (2) analysed to produce national estimates of key population size, HIV prevalence and ART coverage among female sex workers, men who have sex with men, people who inject drugs, and transgender women.

6. How will research products be shared in the community of study?

For instance, will you be providing written or oral layperson summaries for non-academic information sharing? Will study data be made available to institutions in the region(s) of study? The Lancet Global Health *encourages authors to translate the summary (abstract) into relevant languages after paper editing; do you intend to translate your summary?*

Each year, UNAIDS supports national HIV estimates teams to update the Spectrum suite of mathematical models. Countries enter new surveillance data collected over the previous 12 months, and HIV epidemic models are fit to HIV surveillance and demographic data to produce estimates over the full course of the epidemic. Research products from this analysis have been used to create a Key Population Workbook which was introduced into Spectrum to support HIV estimates teams to review and validate their key population data.<br><br>All data collated and analysed for this manuscript is available on Zenodo (link provided in the manuscript). A French abstract will be made available to support community members, national programmers, and academic researchers from French-speaking West and Central African countries.

7. How were individuals, communities, and environments protected from harm?

a) How did you ensure that sensitive patient data was handled safely and respectfully? Was there any potential for stigma or discrimination against participants arising from any of the procedures or outcomes of the study?

This study was a secondary analysis of publicly available data. All data ascertained for this study were aggregate and fully anonymised. There was no risk to individual study participants resulting from this analysis.

b) Might any of the tests be experienced as invasive or culturally insensitive?

This study involved only secondary analysis of existing data; no tests were conducted as part of this study.

c) How did you determine that work was sensitive to traditions, restrictions, and considerations of all cultural and religious groups in the study population?

The manuscript and its conclusions were critically reviewed by all co-authors, several of whom have worked within HIV epidemiology among key populations and alongside key population communities for many years.

d) *Were biowaste and radioactive waste disposed of in accordance with local laws?*

Not applicable

e) *Were any structures built that would have impacted members of the community or the environment (such as handwashing facilities in a public space)? If so, how did you ensure that you had appropriate community buy-in?*

Not applicable

f) *How might the study have impacted existing health-care resources (such as staff workloads, use of equipment that is typically employed elsewhere, or reallocation of public funds)?*

Not applicable

8. Finally, please provide the title (eg, Dr/Prof, Mr/Mrs/Ms/Mx), name, and email address of an author who can be contacted about this statement. This can be the corresponding author.

Name: Mr Oliver Stevens
Email: o.stevens@imperial.ac.uk
